# Editorial: Floral adaptations and pollinator dynamics in a rapidly changing environment

**DOI:** 10.3389/fpls.2025.1756585

**Published:** 2026-01-06

**Authors:** Monika M. Lipińska, Adam P. Karremans

**Affiliations:** 1 Department of Plant Taxonomy and Nature Conservation, Faculty of Biology, University of Gdańsk, Gdańsk, Poland; 2 Centro de Investigación Jardín Botánico Lankester, Universidad de Costa Rica, Cartago, Costa Rica; 3 Instituto de Investigación en Ciencias Naturales y Tecnología (Iarna), Universidad Rafael Landívar, Ciudad de Guatemala, Guatemala

**Keywords:** ecology, floral traits, global change, plant- animal interactions, pollination

Across ecosystems, the delicate synchrony between plants and their pollinators sustains organism abundance and diversity, food webs, and ecosystem resilience. Yet this synchrony is increasingly strained by a rapidly changing environment. Alterations in temperature, precipitation, and land use reshape species distributions, flowering times, and pollinator behaviour. As the pace of change accelerates, the once stable evolutionary partnerships between plants and pollinators are entering a period of unprecedented flux. Understanding the mechanisms, outcomes, and adaptive responses within these systems is therefore among the most urgent challenges in contemporary plant and conservation science.

The Research Topic “*Floral Adaptations and Pollinator Dynamics in a Rapidly Changing Environment”* brings together an interdisciplinary Research Topic that explore how plants and pollinators interact, adapt, and possibly decouple under shifting ecological and climatic conditions. The contributing articles span ecosystems from tropical forests and temperate grasslands to alpine and Arctic regions, providing a broad biogeographic perspective on pollination processes. Together, they reveal how environmental gradients, climate change, habitat fragmentation, and evolutionary history shape floral traits, reproductive strategies, and network dynamics.

## Integrating perspectives from climate gradients to community networks

Several contributions examine how climatic variability influences floral traits and pollination success. van Delden et al. investigate plant traits across New Zealand’s diverse climatic zones, showing that nectar volume, concentration, and sugar composition vary systematically with temperature and rainfall. The study revealed species-specific correlations between plant traits and climate factors in New Zealand tree species. Such physiological plasticity may allow plants to maintain pollinator attraction across changing environments, but it also highlights the vulnerability of specialized mutualisms under shifting conditions.

At the other end of the globe, Khorsand et al. analyze spatial and temporal variation in plant–pollinator networks in the Alaskan Arctic, where short growing seasons and rapidly warming temperatures compress phenological windows significantly. Their results reveal how changes in floral resource availability influence network structure and resilience, providing valuable insights into the mechanisms underpinning Arctic ecosystem responses to climate change.

Complementing these macroecological perspectives, Magray et al. explore how floral traits, pollinator behavior, and breeding systems interact to determine reproductive success in the Himalayan medicinal herb *Phytolacca acinosa* Roxb. Their integrative approach underscores the importance of considering multiple dimensions of plant reproduction when assessing vulnerability to environmental shifts. Similarly, Yan et al. examine scaling relationships between tepal mass and area in the daylily, *Hemerocallis fulva* (L.) L., linking floral symmetry and allometric patterns to developmental and evolutionary constraints that may mediate adaptive responses to changing pollination contexts.

## Vulnerability and resilience under climate change

As the effects of climate change progresses, mismatches between plant phenology and pollinator presence and activity are emerging as a recurrent theme. Watteyn et al. assess this Research Topic in species of the orchid genus *Vanilla* Mill., revealing that wild populations and their pollinators may soon face spatial mismatches due to changing climatic envelopes. The study integrates species distribution modeling with ecological data, offering a predictive framework to guide the crop’s resilience and the conservation of tropical orchids and their specialized pollinators.

In the shrublands of northwestern China, Chen et al. document how habitat fragmentation influences pollinator visitation and reproductive success. Their findings demonstrate that fragmentation not only reduces visitation rates but can also alter selective pressures on floral traits, with implications for long-term evolutionary trajectories. In contrast, Reiter et al. present a hopeful perspective from restoration ecology: they show that co-planting with rewarding species may be an effective approach for improving pollination success of threatened orchids. This work exemplifies how integrating pollinator ecology into conservation design can enhance restoration success.

The interplay between floral polymorphism and reproductive strategy is further explored by Duan et al., who study two color morphs of *Paeonia delavayi* Franch. They reveal contrasting reproductive strategies linked to morph-specific pollinator preferences, suggesting that maintaining phenotypic diversity may serve as a buffer against environmental stressors.

## Expanding methodological and conceptual frontiers

Advancing our understanding of plant–pollinator interactions in changing environments requires innovative tools and perspectives. Several studies in this Research Topic exemplify such innovation. Montero et al. employ pollen metabarcoding to uncover that low-intensive farming practices that promote the presence of common ruderal plants combined with nearby protected forests contribute to maintaining diverse insect communities that provide crucial pollination services. Their results highlight that even in modified habitats, pollinator foraging can connect a wide array of wild and cultivated plants, suggesting that maintaining heterogeneous landscapes may sustain pollination networks.

Meta-analytical and modeling approaches also provide valuable synthetic insights. Alquichire-Rojas et al. compile evidence from multiple studies to evaluate how increased temperatures affect floral rewards and pollinator interactions. Results showed that global warming affects floral rewards in both wild and crop plants, providing insights into the effects of changing climatic conditions on plant-pollinator interactions and pollination service. Similarly, Miao et al. demonstrate that the rise of interannual temperatures leads to more uniform phenological matching between the invasive *Stellera chamaejasme* L. and its pollinators across different elevations. This counterintuitive result underscores the complexity of climate effects: while native species may suffer from mismatches, some invasive plants could gain reproductive advantages under warming conditions.

Expanding the spatial and temporal scope of pollination studies is another emerging frontier. Teng et al. address this challenge by integrating diurnal and nocturnal pollen data into the construction of pollination networks in a subalpine wetland community. Their approach reveals that excluding nocturnal interactions can underestimate network complexity and functional diversity, advocating for more comprehensive sampling frameworks that capture the full temporal dimension of pollination ecology.

## Toward a predictive understanding of floral adaptation

Collectively, the contributions in this Research Topic converge on a central message: the adaptive potential of plants and the resilience of pollination systems depend on complex interactions among organisms. Floral evolution is not a static outcome but a dynamic process continuously shaped by ecological pressures and environmental change.

A key implication is that studying floral adaptations requires moving beyond single-species or single-factor perspectives. Integrative approaches—combining physiology, morphology, genetics, and community ecology—are essential to predict how plant–pollinator systems will respond to future scenarios. Moreover, linking field-based research with experimental manipulations and modeling can help disentangle the feedback between trait evolution and pollination dynamics.

The studies included here also highlight the importance of geographic breadth and methodological diversity. From the Arctic to the tropics, from meta-analyses to metabarcoding, they demonstrate that understanding pollination under global change demands both global coordination and local insight. In doing so, they contribute to a growing framework for anticipating ecological tipping points, identifying conservation priorities, and guiding restoration actions.

As we move deeper into the Anthropocene, the fate of many plant species will depend on their ability to maintain effective pollination. Some will adapt through phenotypic plasticity or shifts in floral reward chemistry; others may rely on generalist pollinators or human-assisted restoration efforts. Yet, as the research in this Topic demonstrates, resilience is not guaranteed for all organisms in an ecosystem. Understanding and mitigating pollination disruption will require collaboration across disciplines, linking plant physiology, evolutionary biology, landscape ecology, and conservation management.

The breadth and depth of work presented in this Research Topic embody this integrative spirit. Together, these studies provide not only snapshots of current challenges but also a roadmap for future inquiry: one that recognizes floral diversity as both a record of past adaptation and a key to sustaining life in an uncertain future.

